# Tracheal intubation for a difficult airway using Airway scope^®^, KingVision^® ^and McGRATH^®^: a comparative manikin study of inexperienced personnel

**DOI:** 10.1186/cc12097

**Published:** 2013-03-19

**Authors:** J Itai, Y Tanabe, T Nishida, T Inagawa, Y Torikoshi, Y Kida, T Tamura, K Ota, T Otani, T Sadamori, K Une, R Tsumura, Y Iwasaki, N Hirohashi, K Tanigawa1

**Affiliations:** 1 Hiroshima University Hospital, Hiroshima-shi, Japan; 2 Hiroshima City Hospital, Hiroshima-shi, Japan

## Introduction

The Airway scope^® ^(AWS), the KingVision^® ^(KV) and the McGRATH^® ^(MG) are new indirect video laryngoscopes designed to facilitate effective and safe tracheal intubation under various conditions. However, there are few comparative studies as for performance in tracheal intubation attempted by inexperienced personnel. The purpose of this study was to evaluate success rates, time to intubation with use of these devices by inexperienced personnel in a simulated manikin difficult airway.

## Methods

Twenty-nine fifth-year medical students with no previous experience in tracheal intubation participated in this study. We used an advanced patient simulator (SimMan^®^; Laerdal Medical, Stavanger, Norway) to simulate normal and difficult airway scenarios including cervical spine rigidity, swollen tongue, and pharyngeal edema. The sequences in selecting devices and scenarios were randomized. Success rate for tracheal intubation, and the time required for visualization of the glottis (T1), tracheal intubation (T2), and inflation of the lungs (T3) were analyzed. Also, numbers of audible dental click during the intubation attempt were recorded. The three different intubation devices were tested in four different scenarios by 29 students.

## Results

All three devices had very high success rates of tracheal intubation (AWS 100%; KV 100%; MG 99%). In the normal airway, T1, T2 and T3 were AWS: 4.4 ± 3.3 seconds, 7.5 ± 3.8 seconds, 11.1 ± 3.8 seconds; KV: 6.9 ± 6.9 seconds, 10.3 ± 7.9 seconds, 14.1 ± 8.3 seconds; MG: 4.6 ± 1.3 seconds, 11.4 ± 5.2 seconds, 16.1 ± 5.4 seconds, respectively (NS). In the three difficult airway scenarios, T1, T2 and T3 were AWS: 5.3 ± 4.7* seconds, 10.4 ± 6.9* seconds, 14.2 ± 6.9* seconds; KV: 10.9 ± 12.8 seconds, 21.5 ± 18.1 seconds, 25.4 ± 18.3 seconds; MG: 13.0 ± 15.3 seconds, 26.2 ± 24.6 seconds, 31.0 ± 24.3 seconds, respectively (*P <0.05 AWS vs. KV and MG). The number of audible dental click sounds with the MG was greater than with the AWS and KV (AWS 5%*; KV 8%*; MG 28%*; *P <0.05 AWS and KV vs. MG).

## Conclusion

The AWS, KV and MG had very high success rates of tracheal intubation and are suitable as intubation devices for inexperienced personnel. In the difficult airway, however, the intubation time with AWS was significantly shorter than with KV and MG. These findings suggested that AWS may be most useful device particularly in difficult situations such as emergency settings. Further studies in a clinical setting are needed to confirm these findings.

